# Changes in the Expression of Bone Morphogenetic Protein 7 and Tamm– Horsfall Protein in the Early Stages of Diabetic Nephropathy

**DOI:** 10.5812/numonthly.2124

**Published:** 2012-03-01

**Authors:** Yanchun Qu, E Du, Yue Zhang, Shengzhi Li, Ruifa Han, Mengsheng Qiu

**Affiliations:** 1 Tianjin Institute of Urology, 2nd Hospital of Tianjin Medical University, Tianjin, China

**Keywords:** Models, Animal, Bone Morphogenetic Protein 7, Diabetic Nephropathies, Uromodulin, NPHS2 Protein, Blotting, Western

## Abstract

**Background:**

Bone morphogenetic protein 7 (BMP7) has been suggested to play a protective role against kidney injury in chronic kidney disease.

**Objectives:**

To identify the critical molecular regulators in the early stage of diabetic nephropathy, we studied the expression of BMP7 and 2 important kidney-specific markers, podocin and Tamm–Horsfall protein (THP).

**Materials and Methods:**

A diabetic nephropathy model was established by intraperitoneally injecting streptozotocin (STZ) in male Kunming mice. Kidney weight index was used as an indicator of early renal injury. Kidney tissue from the diabetic model mice was obtained at 4, 8, and 12 weeks, and total protein was extracted to assess the expression of BMP7, podocin, and THP by western blot analysis.

**Results:**

Diabetic model mice were successfully established, and the kidney weight index of the model animals increased significantly. The expression of BMP7 was significantly downregulated, while the expression of THP was increased in the early stage of diabetic nephropathy. However, the expression of podocin did not change.

**Conclusions:**

Our observations suggested that down-regulation of BMP7 expression and up-regulation of THP expression were early events that occur prior to podocyte injury with the structure protein, podocin spoiled, which further confirmed that BMP7 is a key molecular regulator in the early stage of diabetic nephropathy.

## 1. Background

Diabetic nephropathy (DN) often progressively develops into end-stage renal disease (ESRD) and accounts for a large percentage of ESRD. Bone morphogenetic protein 7 (BMP7) belongs to the TGFβ superfamily. Its expression is particularly important for kidney development (1). In the adult, BMP7 expression is mainly located in the epithelial cells of the glomeruli and distal tubules in the kidney (2). Recently, BMP7 has been shown to be a potential antifibrotic agent (3, 4). A marked contribution by BMP7 in the striking reversal of TGFβ-driven fibrosis has been demonstrated in a mouse model of renal fibrosis (5).

Tamm–Horsfall protein (uromodulin, THP), an 80-kDa glycoprotein, is exclusively synthesized in the thick ascending limb cells of Henle’s loop (TAL) (6). Marked changes in THP synthesis or urinary excretion have been found in pathological conditions (7-9). Therefore, THP is considered useful as a marker of renal disease (10). Podocin is encoded by the NPHS2 gene, and is exclusively expressed in podocytes, specifically at the point in the slit diaphragm that interacts with CD2AP and nephrin (11). Mutations in NPHS2 lead to the development of nephrotic syndrome (12, 13).

## 2. Objectives

The aim of this work was to investigate the expression pattern of BMP7, THP, and podocin in the early stage of DN and determine their roles in renal injury in the early stage of DN.

## 3. Materials and Methods

### 3.1. Diabetic Mouse Model

The 40 male Kunming mice used in this study were housed until 7 weeks of age with free access to standard mouse chow and water. Prior to the intraperitoneal injection of streptozotocin (STZ), all mice were fasted for 12–16 h, and fasting body weight was recorded. Fasting plasma glucose was measured in tail blood with a Grace Track glucometer. A total of 30 mice were given an intraperitoneal injection of STZ (150 mg/kg) dissolved in citric acid buffer solution (pH 4.5); 10 additional mice were used as controls and given an intraperitoneal injection of the buffer solution. The fasting plasma glucose of the model mice was measured 5 days later, and mice with plasma glucose levels greater than 16.0 mmol/L were used for further study as successful diabetic model animals. The diabetic model mice were kept for 4, 8, or 12 weeks, and then the kidneys were dissected and weighted, snap frozen in liquid nitrogen, and stored at -70°C until assay. The kidney weight index was calculated. The use of Kunming mice as a diabetic model and the experiment design were approved by the local animal committee.

### 3.2. Western Blot Analysis

Fifty micrograms of tissue were dissected from each kidney sample and used for total protein extraction. A Bradford protein assay kit (Beijing Bio-Med Co. Ltd.) was used to measure the protein concentration according to the manufacturer’s instructions. Under reducing conditions, 100 µg of total protein were subjected to SDS/PAGE in 12% Bis-Tris-polyacrylamide gels. After electrophoresis, the proteins were transferred to PVDF membranes and the blots were blocked with 5% non-fat milk in TBST buffer prior to incubation with the appropriate primary antibodies. The primary antibodies were prepared in TBST and incubated at 30°C for 2 h. The primary antibodies used to detect BMP7 and podocin (Wuhan Boster Biotechnology Co. Ltd.) were used at a 1:500 dilution and a 1:300 dilution, respectively. The primary antibodies for THP and β-actin were diluted 1:800, and were purchased from Santa Cruz Biotechnology. The blots were then incubated with alkaline phosphatase-labeled secondary antibody (VECTOR laboratories) for 2 h at room temperature. The protein bands were stained with an NBT/BCIP western-blotting detection system. The blots were then re-probed for β-actin as a loading control. The blot results were photographed with a Samsung Digimax i50 camera. The density of the protein bands was quantified using Band Leader software (version 3.0). An average of 3 replicates were used for quantification of the western results.

## 4. Results

### 4.1. Diabetic Mouse Model

A total of 28 model mice were successfully obtained, and they were divided into 3 groups that were kept in the diabetic state for 4, 8, and 12 weeks. Three model mice accidentally died during the experiment. Ultimately, 9, 9, and 7 model mice kept for 4, 8, and 12 weeks, respectively, were used for the analysis. The phenotypic variables of the model mice in different groups were compared with the control animals by ANOVA (Table 1).

**Table 1:** The Phenotypic Characteristics of Control and Diabetic Model Mice at 4, 8, and 12 Weeks a

**Stage**	**Control**	**4 wk**	**8 wk**	**12 wk**
Fasting weight (g)	39.3 ± 2.9 A	25.1 ± 1.3 B	22.6 ± 2.2 B	25.5 ± 2.5 B
Fasting plasma glucose (mmol)	6.8 ± 2.3 Aa	28.1 ± 4.6 B	27.4 ± 8.3 B	16.2 ± 2.4 Ab
Kidney weight index	1.49 ± 0.18 Aa	1.53 ± 0.13 AaBb	1.58 ± 0.11 ABb	1.74 ± 0.17 Bc

^a^The phenotypic measurements were compared by one-way ANOVA. Different letters in the same line indicate significant differences (uppercase letters for P < 0.01, lowercase letters for P < 0.05) between groups.

### 4.2. Western Blot Analysis

**Figure 1:**
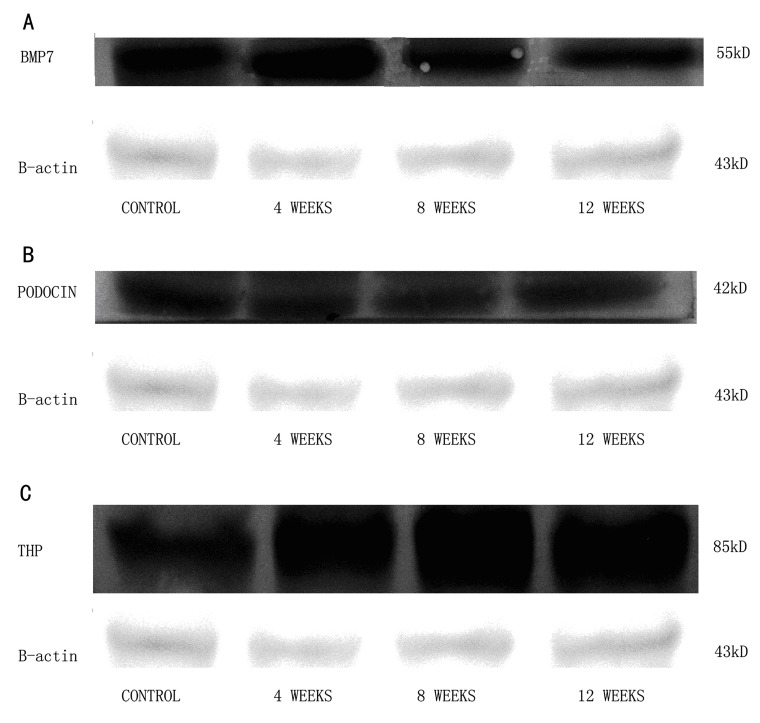
The Western Blot Analysis of the Protein Expression of BMP7 (a), Podocin (b) and THP (c). The Expression of β-actin Was Used as a Loading Control for the Normalization of the Expression of Three Interested Genes.

The results of the western blot for BMP7, podocin, and THP are shown in Figure 1. The expression pattern of the 3 proteins showed that BMP7 expression gradually decreased, while THP increased in the early stage of DN. In contrast, the expression of podocin did not change during this stage.

### 4.3. Quantification of BMP7, Podocin, and THP Protein Expression

To quantify the protein expression of the genes of interest, the intensity of the protein bands were analyzed and quantified using Band Leader software (version 3.0), and the ratio of the band intensity of the genes of interest compared to β-actin was used as the adjusted expression level of the genes of interest. The adjusted expression of the genes of interest is summarized in Table 2. The adjusted BMP7 expression was markedly down-regulated in the early stages of DN. The expression of THP was markedly up-regulated during the first 2 stages, and the expression at 12 weeks was similar to that at 8 weeks. In contrast, the adjusted expression of podocin did not change significantly.

**Table 2:** Quantification of the Expression of BMP7, Podocin, and THP in the Early Stage of Diabetic Nephropathy

**Gene of interest **	**BMP7** **(Intensity/Ratio)**	**Podocin (Intensity/Ratio)**	**THP ** **(Intensity/Ratio)**
Control	187.59/6.47	210.35/6.77	181.20/5.49
4 Wk	193.15/6.43	216.68/6.77	195.14/6.26
8 Wk	178.23/5.56	210.39/6.78	213.70/7.32
12Wk	168.06/5.08	209.68/6.79	210.48/7.17

Gene expression was quantified by an intensity scan using Band Leader software using the ratio obtained after the expression was adjusted relative to the expression of β-actin. An average of 3 replicates were performed for every gene.

## 5. Discussion

In this article, we examined the expression pattern of 3 important proteins, BMP7, podocin, and THP, in the early stage of DN using a diabetic mouse model. BMP7 expression gradually decreased and THP expression significantly increased in the early stage of DN. In contrast, there was no marked change in the expression of podocin protein during this stage. These findings suggested that BMP7 and THP play a role in the early stage of DN, while podocin does not.

The gradually decreased expression of BMP7 in our diabetic mouse model in the early stage of DN is consistent with observations of a DN model in Sprague–Dawley rats, which demonstrated that expression of renal tubular BMP7 and some of its receptors decreased while gremlin, a secreted BMP antagonist, increased (14). However, in the DN mouse model used in our study, BMP7 expression did not decrease more than 50%, as it did in the rat study. The most likely explanation for this difference is that we examined the expression of BMP7 in our mouse model at an earlier stage, less than 12 weeks, than they determined expression (15 weeks or later after the rat model was established). Ignoring the physiological differences between the rat and mouse models of DN, the gradual decrease in BMP7 expression with the progression of DN was consistent. These data suggest that BMP7 is a cellular molecule that plays a key role in adult kidney function, and whose expression was decreased by renal injury in the diabetic condition.

THP is another important protein marker of renal disease. According to our observation, THP expression increased in the early stages of diabetic renal injury, and reached a peak 8–12 weeks after the diabetic mouse model was established. This result is consistent with the belief of some nephrologists who concentrate on the changes in THP expression in the early stages of DN (15, 16). However, it is inconsistent with other studies that focused on the later stages and suggested decreased THP expression in diabetic nephropathy (7, 8). Further work is needed to describe the whole picture of THP expression during the progression of DN.

Podocin is another key molecule in renal disease that has attracted researchers, partly because it is a key structural protein that assembles and stabilizes the slit diaphragm. Recent studies of podocyte-specific genes in proteinuric diseases found that podocin expression varied the most and can be used for molecular discrimination of some renal diseases (17). However, according our observations, there was no change in podocin expression in the early stage of DN. We inferred that the expression of podocin protein differed from podocin RNA expression, and the change in podocin protein expression occurred after down-regulation of BMP7 expression.

In this study, we used kidney weight index as an early predictor of renal injury in early diabetic nephropathy. It is well known that glomerular filtration rate (GFR) is an important indicator of renal function. However, during early renal injury in DN, GFR first increases then decreases. In contrast, reversal of kidney enlargement does not occur. Therefore, kidney weight index should be a more stable indicator of renal injury than GFR, and it is also easier to handle (18, 19).

In conclusion, this study focused on the expression pattern of BMP7, THP, and podocin in the early stage of DN, and marked changes in BMP7 and THP expression were shown. The results suggest that BMP7 and THP are 2 molecules upstream of renal injury in DN, whose regulation should be useful for determining the molecular mechanism underlying the initiation of renal injury due to high plasma glucose. However, the molecular mechanism for the regulation of BMP7 and THP expression is yet unknown. Fortunately, more and more new molecules and tools are being developed with the research advancements in this field, which will be of great help for future studies (20, 21).

## References

[1] Kazama I, Mahoney Z, Miner JH, Graf D, Economides AN, Kreidberg JA (2008). Podocyte-derived BMP7 is critical for nephron development.. J Am Soc Nephrol.

[2] Markic D, Celic T, Spanjol J, Grskovic A, Bobinac D, Fuckar Z (2010). Expression of bone morphogenetic protein-7, its receptors and Smad1/5/8 in normal human kidney and renal cell cancer.. Coll Antropol.

[3] Motazed R, Colville-Nash P, Kwan JT, Dockrell ME (2008). BMP-7 and proximal tubule epithelial cells: activation of multiple signaling pathways reveals a novel anti-fibrotic mechanism.. Pharm Res.

[4] Zeisberg M, Kalluri R (2008). Reversal of experimental renal fibrosis by BMP7 provides insights into novel therapeutic strategies for chronic kidney disease.. Pediatr Nephrol.

[5] Zeisberg M, Hanai J, Sugimoto H, Mammoto T, Charytan D, Strutz F (2003). BMP-7 counteracts TGF-beta1-induced epithelial-to-mesenchymal transition and reverses chronic renal injury.. Nat Med.

[6] Serafini-Cessi F, Malagolini N, Cavallone D (2003). Tamm-Horsfall glycoprotein: biology and clinical relevance.. Am J Kidney Dis.

[7] Lau WH, Leong WS, Ismail Z, Gam LH (2008). Qualification and application of an ELISA for the determination of Tamm Horsfall protein (THP) in human urine and its use for screening of kidney stone disease.. Int J Biol Sci.

[8] Sejdiu I, Torffvit O (2008). Decreased urinary concentration of Tamm-Horsfall protein is associated with development of renal failure and cardiovascular death within 20 years in type 1 but not in type 2 diabetic patients.. Scand J Urol Nephrol.

[9] Akioka Y, Chikamoto H, Horita S, Yago R, Tanabe K, Yamaguchi Y (2009). Screening of vesicoureteral reflux in pediatric patients with kidney transplantation showing non-specific interstitial fibrosis and tubular atrophy with interstitial Tamm-Horsfall protein deposits in protocol allograft biopsy.. Clin Transplant.

[10] Mollsten A, Torffvit O (2010). Tamm-Horsfall protein gene is associated with distal tubular dysfunction in patients with type 1 diabetes.. Scand J Urol Nephrol.

[11] Gerke P, Sellin L, Kretz O, Petraschka D, Zentgraf H, Benzing T (2005). NEPH2 is located at the glomerular slit diaphragm, interacts with nephrin and is cleaved from podocytes by metalloproteinases.. J Am Soc Nephrol.

[12] Philippe A, Weber S, Esquivel EL, Houbron C, Hamard G, Ratelade J (2008). A missense mutation in podocin leads to early and severe renal disease in mice.. Kidney Int.

[13] Sun H, Zhou W, Wang J, Yin L, Lu Y, Fu Q (2009). A novel mutation in NPHS2 gene identified in a Chinese pedigree with autosomal recessive steroid-resistant nephrotic syndrome.. Pathology.

[14] Wang SN, Lapage J, Hirschberg R (2001). Loss of tubular bone morphogenetic protein-7 in diabetic nephropathy.. J Am Soc Nephrol.

[15] Pfleiderer S, Zimmerhackl LB, Kinne R, Manz F, Schuler G, Brandis M (1993). Renal proximal and distal tubular function is attenuated in diabetes mellitus type 1 as determined by the renal excretion of alpha 1-microglobulin and Tamm-Horsfall protein.. Clin Investig.

[16] Catalano C, Torffvit O (1996). Urinary excretion of Tamm-Horsfall protein in normotensive, normo-albuminuric type 1 diabetic patients.. Nephron.

[17] Schmid H, Henger A, Cohen CD, Frach K, Grone HJ, Schlondorff D (2003). Gene expression profiles of podocyte-associated molecules as diagnostic markers in acquired proteinuric diseases.. J Am Soc Nephrol.

[18] Bak M, Thomsen K, Christiansen T, Flyvbjerg A (2000). Renal enlargement precedes renal hyperfiltration in early experimental diabetes in rats.. J Am Soc Nephrol.

[19] Christiansen JS, Parving HH (1982). The relationship between kidney size and function in short-term diabetic patients.. Diabetologia.

[20] Wang S, Hirschberg R (2011). Y-box protein-1 is a transcriptional regulator of BMP7.. J Cell Biochem.

[21] Zouvelou V, Passa O, Segklia K, Tsalavos S, Valenzuela DM, Economides AN (2009). Generation and functional characterization of mice with a conditional BMP7 allele.. Int J Dev Biol.

